# Reduced cognitive ability in people with rheumatoid arthritis compared with age-matched healthy controls

**DOI:** 10.1093/rap/rkab044

**Published:** 2021-06-27

**Authors:** James M Gwinnutt, Task Toyoda, Stephen Jeffs, Emma Flanagan, Jacqueline R Chipping, Jack R Dainty, Eneida Mioshi, Michael Hornberger, Alex MacGregor

**Affiliations:** 1 Centre for Epidemiology Versus Arthritis, Centre for Musculoskeletal Research, Faculty of Biology, Medicine and Health, Manchester Academic Health Science Centre, The University of Manchester, Manchester; 2 Norwich Medical School, University of East Anglia; 3 Rheumatology Department, Norfolk and Norwich University Hospitals NHS Trust; 4 School of Health Sciences, University of East Anglia, Norwich, UK

**Keywords:** rheumatoid arthritis, cognition, memory, attention, executive function, psychology, mental health

## Abstract

**Objective:**

The aim was to compare the cognitive ability of people with RA with healthy controls (HCs).

**Methods:**

People with RA were recruited from the Norfolk Arthritis Register (NOAR), a population-based cohort study of people with inflammatory arthritis. Data on aged-matched HCs (people with no cognitive impairment) came from the comparison arm of The Dementia Research and Care Clinic Study (TRACC). People with RA and HCs performed a range of cognitive ability tasks to assess attention, memory, verbal fluency, language, visuospatial skills, emotional recognition, executive function and theory of mind. A score of <88 on the Addenbrooke’s Cognitive Examination III was considered cognitive impairment. Scores were compared using linear regression adjusting for age, sex, smoking status, education, BMI, anxiety and depression.

**Results:**

Thirty-eight people with RA [mean (S.D.) age: 69.1 (8.0) years; 25 (65.8%) women] were matched with 28 HCs [mean (S.D.) age: 68.2 (6.4) years; 15 (53.6%) women]. Twenty-three (60.5%) people with RA were considered to have mild cognitive impairment [mean (S.D.) Addenbrooke’s Cognitive Examination III: RA = 85.2 (7.4), HC = 96.0 (2.5)]. People with RA had impairments in memory, verbal fluency, visuospatial functioning, executive function and emotional recognition in faces compared with HCs, after adjustment for confounders.

**Conclusion:**

People with RA had cognitive impairments in a range of domains. People with RA might benefit from cognitive impairment screening to allow for early administration of appropriate interventions.

**Key messages**

- In this study, 60.5% of the people with RA had cognitive impairment.
- People with RA had impairments in memory, verbal fluency, executive function and emotional recognition.
- People with RA may require cognitive impairment screening, given the negative consequence on daily life.

## Introduction

RA is a chronic inflammatory condition that results in swollen joints, pain and functional disability. RA is associated with increased risk of other conditions, with some evidence suggesting an association between RA and increased risk of dementia [1], but evidence is conflicting [2]. This may be attributable to confounding factors such as smoking, education and medication [3].

A precursor to dementia is mild cognitive impairment (MCI), involving memory and cognitive difficulties that have not developed into a loss of independence. People with MCI suffer from reduced quality of life [4], reduced functional ability [5] and lower adherence to treatment for other conditions [6]. Evidence suggests that some people with RA might have MCI, because they experience difficulties compared with controls in certain aspects of cognition, such as verbal function, memory and attention [7]. Potential drivers of cognitive impairment in RA could operate through the relationship between RA and cardiovascular disease (itself known to be a predictor of cognitive impairment) [8], an increased occurrence of CNS-related autoantibodies [9], or the influence of depression or anti-rheumatic medication [7]. However, previous studies have been hampered by relatively small sample sizes, inconsistent management of confounding variables and limited range of cognitive domains assessed. Any screening for MCI may need the administration of a large battery of cognitive tests, but at present it is unclear whether administration of such a battery is feasible or necessary.

Therefore, the objectives of this study were to assess the value and feasibility of conducting a range of tests that assess different cognitive domains in people with RA, and to examine whether people with RA exhibit a specific pattern of cognitive impairment compared with healthy controls (HC), after taking into account important confounding factors.

## Methods

The Norfolk Arthritis Register (NOAR) is a prospective inception cohort of people with inflammatory arthritis (and its subset, RA), situated in Norfolk, UK [10]. Recruitment began in 1990, and the inclusion criteria are at least two swollen joints for ≥4 weeks. For the present study, we identified from the NOAR database prospective participants who met the following criteria: (i) age ≥54 years; (ii) RA according to the 2010 ACR/EULAR criteria [11]; (iii) symptom duration ≥1 year; and (iv) no known cognitive impairment, neurological or neurodegenerative disease (such as dementia). These NOAR participants were invited to take part in the study.

Control data came from The Dementia Research and Care Clinic Study (TRACC). TRACC is a transdiagnostic study aiming to recruit and follow longitudinally participants from Norfolk and the neighbouring county of Suffolk with a range of diagnoses (Alzheimer’s disease, MCI, motor neuron disease and Pick’s disease complex) in addition to a cohort of HCs for comparison. Participants were eligible to take part in the HC sample of TRACC if they were: (i) aged between 50 and 75 years; (ii) scored >88 on the Addenbrooke’s Cognitive Examination III (ACE-III; see below for description); and (iii) were not diagnosed with dementia or a current mental health disorder. Furthermore, to be included as controls in the present study, participants had to have no diagnosis of RA and have completed the same cognitive assessments as the NOAR participants.

Both NOAR and TRACC received ethical approval (NOAR: 15/EE/0076; TRACC: 16/LO/1366), and all participants provided written informed consent.

### Cognitive assessments

People with RA and HCs underwent a series of cognitive assessments aiming to assess a range of different cognitive domains. For all scales, higher scores indicate better cognitive function.

- The Addenbrooke’s Cognitive Examination III (ACE-III): cognitive tests assessing five cognitive domains, namely attention, memory, verbal fluency, language and visuospatial skills [12]. A total score of <88 indicates cognitive impairment [13].
- Institute of Cognitive Neurology Frontal Screening (IFS): measure of executive function [14].
- Mini Social Cognition and Emotion Assessment (mini-SEA): faux-pas understanding (theory of mind test) and facial emotional recognition [15].
- Rey Complex Figure Test (RCFT): assessment of visuospatial function, in which participants copy abstract images before later drawing from memory [16].

The participants with RA also completed a feedback form regarding the acceptability of the cognitive tests.

### Covariates

Age, sex, smoking status (current, former or never) and education level [no qualifications, GCSEs or O levels (qualifications at 16 years in the UK), A levels (qualifications at 18 years in the UK) and/or degree] were self-reported by all participants. Height and weight were measured by research staff at assessments, and BMI (in kilograms per square metre) was calculated. Anxiety and depression severity were assessed by the Generalized Anxiety Disorder-7 (GAD-7) [17] and the Patient Health Questionnaire-9 (PHQ-9) [18], respectively.

### Statistical analysis

HCs from TRACC were age matched with the NOAR participants. Demographics, cognitive assessments and feedback were summarized using descriptive statistics, stratified by RA/HC status. Differences in characteristics between people with RA and HCs were assessed using Student’s unpaired *t*-tests or χ^2^ tests, depending on the data. Differences in the cognitive assessment scores between the people with RA and controls were assessed using linear regression, controlling for age, sex, BMI, smoking status, education, anxiety and depression. Given that each cognitive assessment has a different scale, to aid comparability the cognitive assessments were also standardized, and the standardized scores were compared between groups using linear regression (controlling for the same covariates). These standardized mean differences (SMDs) represent the mean difference in standard deviations of the assessment between people with RA and controls. Mean and median substitution was used to impute missing data. Analyses were performed using Stata v.14 (StatCorp, College Station, TX, USA).

### Patient and public involvement

Three patient partners were involved in the design of the study and piloted the assessments to check the acceptability and feasibility for future participants.

## Results

In total, 38 people with RA were recruited to this study [mean age = 69.1 (s.d.) 8.0] years, 25 (65.8%) women, mean symptom duration = 9.8 (S.D. 6.3) years]. These people with RA were matched with 28 HCs from TRACC. There were more women, people of higher BMI, people with lower education and smokers in the RA group compared with the controls. Furthermore, the people with RA had significantly higher anxiety and depression scores compared with the controls (Table 1).

**Table 1:** Demographic characteristics of the people with RA and healthy controls

Variable	People with RA	Healthy controls	*P*-value
*n*	38	28	
Age, mean (s.d.), years	69.1 (8.0)	68.2 (6.4)	0.64^a^
Female, *n* (%)	25 (65.8)	15 (53.6)	0.32^b^
Symptom duration, mean (s.d.), years	9.8 (6.3)	–	–
BMI, mean (s.d.), kg/m^2^	28.3 (5.7)	24.1 (4.4)	0.002^a^
Education, *n* (%)			
No qualifications	17 (44.7)	5 (17.9)	0.07^b^
GCSEs/O levels	6 (15.8)	7 (25.0)	
A levels and/or degree	15 (39.5)	16 (57.1)	
Smoking status, *n* (%)			
Never	17 (44.7)	18 (64.3)	0.12^b^
Former	14 (36.8)	9 (32.1)	
Current	7 (18.4)	1 (3.6)	
Depression mean (s.d.), (PHQ-9, range: 0–27^c^)	6.0 (6.7)	1.1 (1.8)	0.0004^a^
Anxiety mean (s.d.), (GAD-7, range: 0–21^c^)	4.9 (5.5)	0.6 (1.3)	0.0001^a^

a Student’s unpaired *t*-test. ^b^χ^2^ test. ^c^Higher scores indicate worse depression/anxiety. GAD-7 = Generalised Anxiety Disorder-7; GCSE = General Certificate of Secondary Education; PHQ-9 = Patient Health Questionnaire-9.

The cognitive assessment battery was acceptable for the people with RA, taking between 90 and 120 min to complete. One hundred per cent of the people with RA either agreed or strongly agreed that the time available to complete the tests was about right, and 89% agreed or strongly agreed that the amount of paperwork was about right.

People with RA had poorer function on many of the cognitive ability assessments compared with HCs after confounding adjustment, including memory, verbal fluency, language, visuospatial processing (ACE-III), executive function (IFS) and emotional recognition from faces (Mini-SEA) (Table 2). In total, 23 (60.5%) people with RA scored <88 on the total score of the ACE-III, indicating cognitive impairment (compared with 0 HCs, because this was an inclusion criterion for the HC group). There were no significant differences between people with RA and HCs in terms of attention (ACE-III), theory of mind (faux-pas test from Mini-SEA) and visuospatial functioning when measured with the RCFT. When assessing the standardized scores, the largest impairments in cognitive ability between people with RA and controls were seen in the memory and visuospatial components of the ACE-III (memory SMD −0.8, 95% CI −1.2, −0.4; visuospatial SMD −1.0, 95% CI −1.5, −0.5) and in executive function (IFS; SMD −1.1, 95% CI −1.5, −0.7).

**Table 2:** Scores on the cognitive assessments, stratified by RA/healthy control status

Cognitive assessment (range)^a^	People with RA, mean (S.D.)	Healthy controls, mean (S.D.)	Adjusted mean difference (95% CI)^b^	Adjusted SMD (95% CI)^c^	*P*-value of adjusted mean differences
ACE-III					
Total (0–100)	85.2 (7.4)	96.0 (2.5)	−7.0 (−9.6, −4.4)	−0.9 (−1.2, −0.6)	<0.0001
Attention (0–18)	16.5 (1.9)	17.7 (0.5)	0.0 (−0.7, 0.7)	0.0 (−0.4, 0.4)	0.952
Memory (0–26)	19.8 (4.0)	24.8 (1.6)	−3.3 (−5.0, −1.5)	−0.8 (−1.2, −0.4)	<0.0001
Verbal fluency (0–14)	9.9 (2.6)	12.1 (1.3)	−1.7 (−2.9, −0.5)	−0.7 (−1.2, −0.2)	0.007
Language (0–26)	24.6 (1.7)	25.6 (0.6)	−0.7 (−1.5, −0.01)	−0.5 (−1.0, −0.01)	0.047
Visuospatial (0–16)	14.4 (1.5)	15.8 (0.5)	−1.3 (−2.0, −0.6)	−1.0 (−1.5, −0.5)	<0.0001
IFS (0–30)	20.4 (3.4)	25.3 (2.0)	−4.1 (−5.7, −2.6)	−1.1 (−1.5, −0.7)	<0.0001
Mini-SEA					
Facial recognition (0–15)	10.3 (1.1)	11.2 (0.9)	−0.8 (−1.3, −0.2)	−0.7 (−1.2, −0.2)	0.005
Faux-pas (0–15)	14.2 (1.2)	14.0 (1.2)	0.4 (−0.3, 1.1)	0.3 (−0.2, 0.9)	0.232
RCFT					
Copy (0–36)	30.2 (5.1)	33.3 (2.9)	−1.3 (−3.8, 1.1)	−0.3 (−0.8, 0.2)	0.277
Recall (0–36)	15.2 (7.9)	18.1 (5.3)	−0.8 (−4.9, 3.3)	−0.1 (−0.7, 0.5)	0.711

a Higher scores indicate better function. ^b^Mean difference from linear regression, controlling for age, sex, BMI, smoking status, education level, depression (PHQ-9) and anxiety (GAD-7). ^c^Each cognitive variable is standardized; the coefficients represent the number of standard deviations difference between people with RA and controls, adjusted for the same confounders above. ACE-III = Addenbrooke’s Cognitive Examination III; GAD-7 = Generalised Anxiety Disorder-7; IFS = Institute of Cognitive Neurology Frontal Screening; Mini-SEA = Mini Social Cognition and Emotion Assessment; PHQ-9 = Patient Health Questionnaire-9; RCFT = Rey Complex Figure Test; SMD = standardized mean difference.

## Discussion

This study demonstrates that performing cognitive test batteries is feasible for people with RA, and these particular assessments are acceptable to participants (e.g. ACE-III; judged based on the participant evaluation). Furthermore, there is a clear signature of reduced cognitive ability in this group compared with controls. In total, 60.5% of the people with RA scored below the ACE-III cut-off, indicating cognitive impairment. When investigating the individual dimensions, people with RA had impaired memory, verbal fluency, executive function and emotional recognition compared with HCs. This was independent of confounding factors such as age, anxiety, depression, education and body mass. People with RA had impaired visuospatial functioning compared with HCs as measured by the ACE-III, but not with the RCFT. This could be attributable to differences in difficulty of the tasks, whereby the ACE-III contains a free-recall element (clock drawing), whereas the RCFT involves copying (and later recalling) a pre-specified abstract design. Lastly, there were no differences between the groups in terms of attention and theory of mind.

A systematic review of studies using the ACE reported a cut-point of <88 for cognitive impairment, indicating that a majority of people (60.5%) with RA in this cohort have some cognitive impairment [13]. This is higher than found in some previous studies (e.g. Shin *et al.* [8]: 31%; Appenzeller *et al.* [19]: 30%) but in line with others (e.g. Vitturi *et al.* [20]: 72.4%), although these other studies used different criteria for cognitive impairment, indicating the need for an agreed definition of cognitive impairment in RA [8, 19, 20]. This high prevalence of people with RA scoring <88 on the ACE-III (indicating cognitive impairment) is important because many people with MCI later develop dementia: in one study, 59% of 54 people with multi-domain MCI later developed dementia [21]. This finding, alongside studies indicating that RA is a risk factor for dementia [1], indicates the need for MCI and dementia screening in RA. In this way, early interventions with the potential to reduce future dementia risk can be instituted, in addition to support for patients and families in daily management when dementia is present. The specific areas of cognitive impairment in RA shown in the present study (memory, verbal fluency, executive function and emotional recognition) have been reported in RA in other settings, summarized in a recent systematic review [7]. Furthermore, emerging evidence of a similar pattern of cognitive impairment in other rheumatic diseases has been reported [22, 23].

Our findings suggest that targeted cognitive screening is potentially needed as part of routine practice to detect clinically relevant cognitive changes in RA and other inflammatory diseases. In a clinical setting, it is noteworthy that cognitive assessments could be delivered remotely, which might be an important component of the future management of people with rheumatic diseases. These findings also highlight the need for a more detailed understanding of the risk factors leading to cognitive impairment in this group, in order to guide strategies for prevention and treatment. This includes, for example, the contribution of exposure to anti-inflammatory drug treatments, lifestyle factors (e.g. diet, smoking and alcohol) and related conditions, including pain, anxiety and depression. Furthermore, future research should address the relative contributions of physical and cognitive factors to functional ability in RA [20, 24].

Our study has a number of strengths. We have examined a large array of cognitive assessments. The study was nested in a population-based cohort, allowing the selection of representative cases of RA. However, our participants were volunteers, and it is possible that concern over their cognitive ability might have motivated them to take part. No additional clinical or laboratory testing was performed as part of this study to investigate changes in cognition further. Furthermore, the ACE cut-off for MCI defines cognitive impairment associated with increased risk of dementia, and people below this threshold might require further assessment and monitoring [13]. Given that RA is thought to be a risk factor for dementia, perhaps a less severe cut-off should be used when screening this group, which could be addressed in future research. We excluded younger people with RA (<54 years) in order to maximize the power to detect cognitive impairment in RA. However, this means that the results are not generalizable to younger people with RA. The limited sample size meant that we could not test the association between clinical factors (e.g. co-morbidities, medications) and cognitive impairment in RA.

In conclusion, this study shows that people with RA have lower cognitive ability across a range of domains compared with HCs. Given the large detrimental effect that cognitive impairment can have on function and quality of life and the feasibility and acceptability of performing cognitive assessments for people with RA, screening of cognitive ability might be warranted in this clinical group.

## Data Availability

De-identified data are available upon reasonable request to the study team.
